# Hidden faces, altered perceptions: the impact of face masks on interpersonal perception

**DOI:** 10.3389/fpsyg.2023.1203442

**Published:** 2023-06-21

**Authors:** Shuai Wang, Chengyang Han, Zihan Sang, Xuhui Zhang, Shitao Chen, Haoran Wang, Gang Wang, Yiqian Xu, Xue Lei, Jixu Chen

**Affiliations:** ^1^Department of Psychology, College of Education, Hangzhou Normal University, Hangzhou, China; ^2^Zhejiang Philosophy and Social Science Laboratory for Research in Early Development and Childcare, Hangzhou Normal University, Hangzhou, China; ^3^School of Business Administration, Zhejiang University of Finance and Economics, Hangzhou, China; ^4^Chinese Education Modernization Research Institute of Hangzhou Normal University (Zhejiang Provincial Key Think Tank), Hangzhou, China

**Keywords:** face mask, face recognition, facial attractiveness, speech perception, trustworthy judgment

## Abstract

The pandemic has made wearing masks commonplace, prompting researchers to investigate their effects on interpersonal perception. Findings indicate masks obstruct face identification and expression recognition, with lower face cues being most affected. When judging attractiveness, masks can enhance the appeal of less attractive faces, but reduce the appeal of more attractive faces. Trust and speech perception outcomes are inconclusive. Future studies could focus on individual differences in how masks influence our perception of others.

## 1. Introduction

Interpersonal perception has been a popular research topic since the last century. With the outbreak of the COVID-19 pandemic, researchers began to focus on the effects of wearing face mask on interpersonal perception. Studies have found that wearing face mask has a significant impact on interpersonal perception, such as face identity recognition, facial expression recognition, attractiveness judgments, trustworthy judgments and speech perception (e.g., Carbon, 2020; Carragher and Hancock, 2020; Olivera-La Rosa et al., 2020). This paper systematically review the effect of face masks on the face processes of interpersonal perception and explore the reasons of impacts. In addition, the paper analyzes the limitations of the current research and provides a prospect for future research directions in this field.

### 1.1. Literature search process

Firstly, this critical review combines the keywords “face mask/mask” with “face recognition/identity recognition/expression recognition/social judgment/facial attractiveness/speech perception/trustworthy judgment/interpersonal perception/child/elder/patient” to search in the Web of Science and Google Scholar databases. The initial search was conducted on November 2021, and the second search was performed on February 2023. A total of 136 articles and 2 conference reports were obtained. These articles and meeting reports were subsequently included in the critical review process. Additionally, it should be noted that the content related to research on transparent masks (e.g., “hello mask”) was supplemented with relevant information obtained through Google search and the APA reference format was retrieved from Google Scholar.

## 2. The influence of mask on face identity recognition and facial expression recognition

Face perception is an important component of the process of interpersonal perception. Researchers mainly focused on the impact of masks on two types of face perception: face identity recognition and facial expression recognition.

### 2.1. Mask and face identity recognition

Several studies have found that individuals’ face recognition performance was weakened when stimuli face wearing surgical face masks. Carragher and Hancock (2020) employed the classic Garner paradigm (Garner, 1976), and found that participants showed worse performance in the mask condition (stimuli face wearing masks) than that in the control condition (stimuli face without masks). Freud et al. (2020) discovered the same results using the Cambridge Face Memory Test paradigm. These studies consistently suggest that wearing a face mask has a significant negative impact on facial recognition in adults.

“Regarding children, living in face mask-wearing environments may impact their development of face perception.” Studies have shown a tendency in early human life to detect coarse face schemes (e.g., two eyes above the mouth), which may be impeded in situations where the lower face cannot be recognized (Rosa-Salva et al., 2010; Reid et al., 2017; Taubert et al., 2017; Buiatti et al., 2019; Versace et al., 2020). Recently, Ferrari et al. (2021) proposed a hypothesis from the perspectives of neuroanatomy and neurophysiology that living in an environment with prolonged use of face masks will alter the synaptic plasticity that facial feature recognition relies on. Children who live in mask-wearing interactions environments for long periods may impact the critical period of developing their facial identity recognition abilities.

### 2.2. Mask and facial expression recognition

Recently, the impact of wearing face mask on emotion recognition has become a research hotspot. Theoretically, there are three ways to identify emotions, and all of them are impeded by face mask: Firstly, visual information from the stimulus face can be matched with the emotional representation stored in the perceiver’s memory. Perceiver can identify the stimulus face’s emotion based on this matching process. Secondly, perceivers can recognize the emotions of the stimulus face better through imitation (Wood et al., 2016). However, face masks reduce the visual information conveyed by the face, also interfering with the perceiver’s imitation of the stimulus face’s emotion. Thirdly, people can rely on contextual information to recognize facial expressions (Hassin et al., 2013). Nonetheless, this type of recognition is influenced by the perceiver’s stereotypes and expectations of the emotional context, which may cause target facial expressions to be misidentified (Maringer et al., 2011).

Those points theoretically explain the negative impact of face masks on facial expression recognition. Carbon (2020) found that wearing face mask made some facial expressions more likely to be misidentified (compared to not wearing face mask), such as happy, sad, angry and disgust. Langbehn et al. (2022) found that face masks had a large impact on the recognition of happy and disgusted facial expressions, but had less influence on the recognition of angry and surprised facial expressions. Additionally, the recognition of fearful and neutral facial expressions was not affected by the presence of face masks (Carbon, 2020; Langbehn et al., 2022), though Marini et al. (2021) found neutral faces may be misinterpreted as sad facial expressions with face masks. That evidence suggests face masks affect emotion recognition differently, which means different emotions may transmit signals at different positions of face (Langbehn et al., 2022), and facial expressions that are recognized based on the lower face are more affected by face masks. Super-recognizers who process face rely on the upper face (i.e., eye range, Tardif et al., 2019) are less impacted by face mask on facial expression recognition (Noyes et al., 2021). Individuals living in mask-wearing interactions environment for long periods may develop behavioral adaptations that rely more heavily on visual cues from the eyes for emotional information (Sheldon et al., 2021).

It should be noticed that the face stimuli employed in the studies mentioned above were various, which may cause different results. For example, Carbon’s (2020) used static image materials, while Langbehn et al.’s (2022) used dynamic video materials (where facial emotions transitioned from neutral to other emotions). Further research may consider the potential existence of the benefits of facial motion.

Furthermore, the intensity of the facial expressions perception is also impacted by face masks. Wearing face masks reduces the positivity conveyed by reward smiles, eliminates the perceived dominance of dominant smiles, and slightly reduces the comforting signal (non-threatening signals) conveyed by affiliation smiles (Langbehn et al., 2022). Sheldon et al. (2021) found that after wearing face masks, fake smiles were recognized as neutral emotions, while real smiles could still be correctly recognized.

The negative impact of face masks on emotional recognition also exists in children. Schneider et al. (2022) found the recognition accuracy of 3–5-year-old children for three facial expressions (happiness, anger, and sadness) were impacted by face mask. Roberson et al. (2012) found that as children aged their accuracy in recognizing unmasked facial expressions gradually increased, but their accuracy in recognizing masked facial expressions remained at a lower level.

It is important to note that the child participants in these studies only live in mask-wearing interaction environments for short periods. However, some infectious diseases have a prolonged impact (e.g., COVID-19), resulting in longer periods of mandatory mask-wearing. From the perspective of emotional recognition development, long-term living in mask-wearing interactions environment may have a significant negative impact on children’s emotional perception abilities (Nelson et al., 2019). Children perceive attitudes and form self-concepts through learning and imitation of others’ expressions (Braadbaart et al., 2014; Paracampo et al., 2017). However, relying exclusively on the eye range may impact the imitation of facial expressions. In future research, two aspects could be considered: First, examining particularly how face mask affects children’s development of emotion recognition at various stages. Second, explore how living in mask-wearing interactions environment for lengthy durations affect children’s categorization and decoding of facial expressions.

## 3. Mask and social judgment

In this section, we will discuss how face mask influence social judgment of faces. Previous works mainly focused on attractive judgment and trustworthy judgment.

### 3.1. Mask and facial attractiveness

Judgments of face attractiveness are frequently present in interpersonal perception (Little et al., 2011; Ying et al., 2019). In the context of the pandemic, researchers have begun to focus on the effect of face masks on facial attractiveness. There are multiple factors in the effect of face masks on facial attractiveness. Miyazaki and Kawahara (2016) proposed a model called the “sanitary-mask effect.” This model comprehensively explains the impact of face masks on the perception of facial attractiveness and is divided into the following two factors:

The first influencing factor is the occlusion effect. Research has shown that asymmetric facial contours and distorted facial features often reduce facial attractiveness (Rhodes et al., 1998 1999; Scheib et al., 1999; Little and Jones, 2003). Similarly, acne and scars can also lessen facial attractiveness (Jaeger et al., 2018). From the perspective of facial symmetry, face masks conceal the aforementioned unattractive features, enhancing facial attractiveness. However, this theory does not apply to all faces. Miyazaki and Kawahara (2016) discovered that face masks can increase the facial attractiveness of low-attractiveness (low symmetry) faces. On the other hand, they can lessen the facial attractiveness of medium and high-attractiveness faces. Moreover, the positive effect of the masking effect on low-attractiveness faces is significantly higher than the negative effect on medium and high-attractiveness faces. Recently, Kamatani et al. (2021) discovered the same results using a similar paradigm. They explained that face masks enhance the symmetry of low-attractiveness faces by covering unfavorable features, thereby increasing their facial attractiveness. Conversely, medium and high-attractiveness faces often have smooth skin and no distorted facial features. Face masks cover these favorable features, which reduces their facial attractiveness.

The second influencing factor is the unhealthy prime. Face masks are often associated with disease, so they may leave a negative impression of the wearer’s health condition (unhealthy prime) on the perceivers. According to a previous study, facial health is positively correlated with facial attractiveness (Jones et al., 2004), thus making the masked face unattractive. Before the COVID-19 pandemic, Miyazaki and Kawahara (2016) discovered that both low and high-attractiveness faces were assessed as having inferior health conditions when wearing face masks. Moreover, when the lower face is covered by an object unrelated to health (e.g., notebook and paper), the unhealthy prime disappears, indicating that the type of object covering the face can affect the unhealthy prime. Interestingly, Kamatani et al. (2021) found that health ratings of mask-wearing faces have improved after the outbreak of COVID-19 (although faces wearing masks were still considered less healthy than faces without masks). This may be due to a shift in people’s interpretation of the social information conveyed by face masks after the pandemic. During the pandemic, wearing face masks was not only related to personal health but also to protecting community members, preventing the spread of the COVID-19 virus, and adhering to social norms (Davis et al., 2021; Zhao and Knobel, 2021). These studies consistently demonstrate that the disease symbolism conveyed by masks leads to a decrease in facial attractiveness, but with the change in people’s attitudes, this trend begins to reverse.

In general, the hygiene mask model can comprehensively explain the impact of face masks on facial attractiveness perception, but the interaction between the occlusion effect and unhealthy prime is not clear. In addition, specific clues that humans use to judge facial attractiveness not only include symmetry mentioned in previous studies but also average (Langlois et al., 1994; Rubenstein et al., 2002; Baudouin and Tiberghien, 2004; Valentine et al., 2004) and sexual dimorphism (Perrett et al., 1998). Future research could focus on the role of average and sexual dimorphism. For example, previous research has shown that the closer a male face is to the average, the more attractive it is perceived by others (Little and Hancock, 2002). It is not clear whether this theory also applies to faces wearing masks.

Based on previous studies, this paper also raises assumptions: First, the more average the upper face, the more masculine the male face (and the more feminine the female face), the more positive the attractiveness assessment will be. Second, the effect of face masks on the attractiveness of individual faces could applies to group faces. Specifically, face masks enhance the overall attractiveness of low-attractiveness group faces and reduce the overall attractiveness of high-attractiveness group faces. If these assumptions are correct, they will supplement and refine the impact of face masks on facial attractiveness judgments, which has practical implications.

It is noteworthy that the impact of face masks on facial attractiveness has extended to daily work. For example, Wu et al. (2021) revealed that face masks can enhance the perceived attractiveness of employees with average looks, thereby improving customer satisfaction. But still, they can lessen the perceived attractiveness of employees with appealing faces, which will lower consumer satisfaction. The results also suggest that customers’ perception of employee facial attractiveness plays a mediating role between employee mask-wearing and customer satisfaction. These findings indicate that face masks can create a more equitable competitive environment between employees with average facial attractiveness and those with high facial attractiveness, thereby minimizing the impact of their actual attractiveness on customer satisfaction.

### 3.2. Mask and trustworthy judgment

In interpersonal communication, judgments of trustworthiness in others often exist in the later stages of interpersonal perception. Currently, there is no unified conclusion regarding the impact of face masks on trustworthy judgment. Olivera-La Rosa et al. (2020) found that face masks can increase trustworthiness in interpersonal communication, which was replicated (Oldmeadow and Koch, 2021). However, Malik et al. (2021) found that face masks have a negative impact on trustworthy judgment in interpersonal communication, which contradicts the former conclusion. It is worth noting that the following factors may cause inconsistent research findings: (1) Different task paradigms. Olivera-La Rosa et al. (2020) and Oldmeadow and Koch (2021) asked participants to rate the level of trust directly on images of faces with or without masks. In Malik et al.’s (2021) study, experimenters either wore masks or no masks, providing economically relevant advice. Trust is judged by the ratio of participants following the advice. (2) Varied presentation of masked faces. In the experiments conducted by Olivera-La Rosa et al. (2020) and Oldmeadow and Koch (2021), facemasks were digitally overlaid on static facial images. They are static picture stimuli. On the other hand, in Malik et al.’s (2021) study, the experimenter provided economic decision-making advice to the participants via video calls while wearing facemasks. They are dynamic natural stimuli. (3) Different approaches to handling additional variables. In the experiments by Olivera-La Rosa et al. (2020) and Oldmeadow and Koch (2021), the facial materials used consisted of neutral faces, excluding the additional variable of facial emotions. However, in Malik et al.’s (2021) experiment, due to the presentation of masked faces in a natural and dynamic context, it was not possible to completely eliminate the additional variable of facial emotions.

In addition, the impact of face masks on trust is also moderated by the following factors:

#### 3.2.1. Personal normative beliefs about wearing masks

During the COVID-19 pandemic, although wearing masks has become a new norm (Bicchieri et al., 2021), different populations have different personal normative beliefs about mask-wearing. Personal beliefs and attitudes towards face masks (such as beliefs about the effectiveness of face masks, and feelings of aversion towards being forced to wear face masks) can indirectly affect individuals’ trust (Bir and Widmar, 2021; Taylor and Asmundson, 2021). Malik et al. (2021) showed that low-belief individuals were less likely to follow the advice of mask-wearers compared to high-belief individuals. In contrast, there was no significant difference in the compliance rate between these two groups with the advice for experimenters without face masks. These results suggest that personal normative beliefs about wearing masks play a crucial role in the impact of face masks on interpersonal trust.

#### 3.2.2. Individual psychological stress

During infectious disease outbreaks, particularly the COVID-19 pandemic, there is an increase in psychological stress among the general population (Rodríguez-Rey et al., 2020; Rossi et al., 2020). This effect is even greater in groups with higher health risks (Quittkat et al., 2020; Iasevoli et al., 2021).

Previous studies consistently demonstrate that psychological stress has an indirect impact on the effect of masks on interpersonal trust. Biermann et al. (2021) found that in groups with higher levels of psychological stress, negative biases induced by face masks are stronger and interpersonal trust is subsequently reduced. Similarly, Grundmann et al. (2021) discovered that face masks reduce interpersonal trust in older groups with higher health risk stress.

#### 3.2.3. Individuals’ interpretation of the social messages contained in the face masks

Product symbolism suggests that the image of a product contains social information related to the product (Allen, 2002), and people tend to associate themselves with it (Newman et al., 2011). One study showed that the outbreak of the COVID-19 pandemic led to a rapid increase in public face mask usage (Feng et al., 2020), which was previously used almost exclusively by medical professionals. Doctors and nurses were ranked as the most trusted professions (Brenan, 2018). Based on this, Klucarova (2022) speculates that the social information related to medical professionals included in face masks, especially disposable surgical masks, may increase the public’s trust in mask wearers. This speculation provides a new perspective for the study of face masks and trust, but it needs to be confirmed by further research.

Nowadays, the social messages contained in face masks also include the beneficial policies announced by the government, communities, and schools. For instance, Davis et al. (2021) observed that American college students’ interpersonal trust scores considerably increased when they wore face masks and adhered to mask norms in daily contact. Another study showed that in China, face masks have become a symbol of mutual protection and social compliance among community members, and public acceptance of face masks is high (Zhao and Knobel, 2021). These studies have all demonstrated that the positive policy information contained in face masks can increase the public’s trust in mask wearers.

#### 3.2.4. Interpretation of face masks with cultural symbols by individuals

Typically, products with cultural symbols symbolize social identity (e.g., national flags and sports team logos). Research under social identity theory suggests that exposure to and sharing of social identity increases helping behavior and trust among individuals within the same social group (Levine et al., 2005). This result also applies to face masks. For example, Perach and Limbu (2022) used cultural masks (face masks with cultural symbols representing unity) as experimental materials and found that cultural masks significantly increased facial trust ratings. This finding offers helpful suggestions for interpersonal communication during and after pandemics.

In general, the impact of face masks on trust is dynamic and complex, influenced by various factors such as the perception of the wearer and the mask itself. Currently, there is no unified research conclusion.

## 4. Mask and speech perception

In interpersonal perception, the influence of face masks is not limited to the visual level, but also extends to the auditory level, as face masks can affect speech perception.

There is no consensus on the impact of face masks on speech perception. Some studies suggest that face masks impede speech perception (Winch et al., 2013; Wittum et al., 2013), while others show that face masks facilitate it (Mendel et al., 2008). Other studies report that face masks do not affect speech perception (Radonovich et al., 2009; Thomas et al., 2011; Atcherson et al., 2017).

Scholars who support that face masks impede speech perception argue that face masks have two disadvantages in daily speech communication. Firstly, face masks cover the speaker’s mouth, which hides some visual cues from the listener. Secondly, face masks change the sound signal (Saeidi et al., 2016; Saigusa, 2017), reducing language transmission by 3 to 4% (Palmiero et al., 2016). On the other hand, those who believe that face masks enhance speech perception suggest that wearing face masks activates two mechanisms: Firstly, the Lombard effect occurs among speakers, which means that when noise or interference occurs during the conversation, speakers raise their pitch, slow down their speech rate, and increase the clarity of their speech (Lombard, 1911; Asadi et al., 2020). According to research findings, speakers have difficulty suppressing the Lombard effect (Pick et al., 1989), which is an involuntary reflection generated by the speaker’s inability to hear their voice (Junqua, 1993), even in non-interactive environments (Egan, 1972). Secondly, according to the “Hypo-and Hyper-Articulation” theory (Lindblom, 1990), speakers wearing face masks can assess the comprehensibility of their own speech content in real-time. They can also actively control the clarity of their speech to adapt to the interactive environment.

After reviewing previous studies, we have identified three possible reasons for the inconsistent conclusions in this field. Firstly, the material differences in face masks may affect speech recognition accuracy differently (Palmiero et al., 2016; Corey et al., 2020). Secondly, various approaches to handling background noise in studies may affect the results, as the interaction of different background noises with audiovisual cues may vary. Thirdly, few studies guide speakers on which language style to use. Studies have shown that speakers adjust their language style based on the speaking context without explicit guidance (Mendel et al., 2008), which can further affect listeners’ speech perception (Cohn et al., 2021). The following is the research basis for proposing these reasons:

### 4.1. Types of masks

Previous studies have focused on the suppression of certain wave frequencies by different types of face masks. These studies have consistently shown that face masks primarily attenuate sounds above 1 kHz. Different types of face masks have varying degrees of impact on the attenuation (Palmiero et al., 2016; Corey et al., 2020) and signal directivity (Pörschmann et al., 2020) of high-frequency sounds. For instance, N95 masks attenuate sounds above 3 kHz, while surgical and cloth masks only attenuate sounds above 5 kHz (Magee et al., 2020), with little effect on the accuracy of low-frequency speech recognition (Atcherson et al., 2017).

### 4.2. Background noise

There aren’t many studies on the impact of face masks on speech perception when background noise is present, but the findings consistently show that background noise affects the perceiver’s perception of the speaker’s speech. One study discovered that wearing face masks resulted in a 30 to 35% decrease in speech intelligibility compared to a control group without face masks in the presence of background noise (Hampton et al., 2020). Brown et al. (2021) further examined the degree to which three types of masks (surgical, cloth, and transparent masks) and three levels of background noise (0 dB SNR, −5 dB SNR, −9 dB SNR) affect speech clarity. Results showed that under no background noise conditions (0 dB SNR), all types of face masks have minimal effect on speech intelligibility. However, as background noise increased to moderate (−5 dB SNR) and high levels (−9 dB SNR), face masks significantly reduced speech clarity. Transparent and cloth masks had the greatest impact on speech clarity, while surgical masks had the least impact. Also, in this study, speakers felt that it required more effort to communicate when wearing a face mask. This effect was most noticeable in moderate and high levels of background noise.

### 4.3. Language style

In interpersonal communication, speakers adjust their language style according to the situation, and this phenomenon also exists under mask-wearing conditions (Mendel et al., 2008). Based on this, some studies have begun to focus on the impact of face masks on speech perception under different language style conditions, with consistent results. Specifically, Yi et al. (2021) found that the “clear” language style (making an effort to clearly convey sentences to others) enhanced the acoustic signal under mask-wearing conditions. Speakers intentionally slowed down speech and amplified tone to make speech clearer and more understandable (Lombard effect), but this also required more vocal effort. Additionally, Cohn et al. (2021) revealed the same results using a similar paradigm. This study also found that speeches with face masks were more difficult to understand than those without face masks under the “positive-emotional” language style (simultaneously maintaining a smile and expressing positive emotions while uttering sentences) condition. Moreover, there was no difference observed under the “casual” condition (speaking sentences in a natural and casual manner). From an overall perspective, these findings indicate that the impact of face masks on speech perception in various language styles is predominantly positive. Face masks showed only negative effects on positive-emotional language style.

Overall, in addition to audio-visual cues, the impact of face masks on speech perception is also influenced by background noise, mask type, and language style. Although there is no unified conclusion, some studies have shown that speakers intentionally improve speech clarity and understandability. This suggests that we may be able to gradually overcome the negative effects of masks on speech perception. Notably, a study has started to focus on the effect of face masks on speech perception in online communication (Giovanelli et al., 2021), which has high ecological validity. Specifically, researchers conducted a study based on video calls. The results showed that when speakers turned off their cameras (black screen state) or wore face masks (camera on), it reduced their performance and speech perception.

## 5. Discussion

In summary, the impact of face masks on various aspects of interpersonal perception varies. In terms of face recognition, face masks hinder face identification and weaken facial emotion recognition. Emotions that rely on the lower half of the face for signaling are more affected by face masks. In terms of social judgment of faces, face masks increase the attractiveness of faces with lower levels of attractiveness while decreasing the attractiveness of faces with medium and high levels of attractiveness. Regarding trustworthy and speech perception, there are no unified conclusions. Studies on interpersonal perception of masks are at a preliminary stage and need to be further studied.

Most research chose static images as facial materials, with only a few studies using dynamic facial video materials (Miyazaki and Kawahara, 2016; Carbon, 2020; Carragher and Hancock, 2020; Freud et al., 2020; Olivera-La Rosa et al., 2020; Marini et al., 2021; Langbehn et al., 2022). In comparison to static images, video materials have higher ecological validity. The utilization of different types of facial materials may lead to variations in research results, and further research needs to be aware of the potential for the benefits of facial motion. Additionally, most images and videos used in previous studies were sourced from facial databases or temporary recordings of actors (Olivera-La Rosa et al., 2020; Marini et al., 2021; Langbehn et al., 2022), limiting the relevance of the conclusions drawn to actual life. For instance, the emotional materials employed in previous studies were often the faces of trained actors, with emotional expressions biased toward extremes (Carbon, 2020; Marini et al., 2021; Langbehn et al., 2022). In the natural environment, however, perceivers often deal with imperceptible and subtle expressions. Consequently, the impact of face masks on emotion recognition may be more significant in daily life. Furthermore, the majority of earlier studies only placed face masks on facial images without accounting for breathing difficulties or speech impediments that may result from mask use in normal life (Miyazaki and Kawahara, 2016; Olivera-La Rosa et al., 2020; Marini et al., 2021). Future studies are recommended to use natural experiments to explore these research questions.

Mask type has been shown to have a differential effect on speech perception. However, in some aspects, particularly on facial attractiveness and trustworthy judgments, future researchers that want to validate the results of previous studies need to use mask materials that are as close to previous studies as possible. This is because the difference in face masks may affect people’s unhealthy initiation and the trust in health care professionals contained in the face masks. Researchers have focused on transparent masks in addition to certified protective masks like N95 and medical surgical masks. Previous studies have shown that transparent masks can benefit individuals with hearing loss (Atcherson et al., 2017) and promote relationships and trust between medical staff and patients (Kratzke et al., 2021). However, the types of transparent masks used in these studies are different, and their effectiveness of protection against viruses still needs to be examined. It is worth mentioning that some researchers have developed transparent masks that can effectively organize viruses (Fortunato, 2020; He et al., 2020). However, research on these transparent masks is still in its early stages and has not yet been widely used by the public.

Face masks contain a wealth of social and cultural information, including their colors, patterns, and other design elements, which can have an impact on interpersonal perception. For example, one study indicated that people in Japan who wore black masks (symbolizing pollution) were perceived as more negative compared to those wearing white masks (symbolizing purity; Kamatani et al., 2021). Another study discovered that face masks with smiling expressions could have a calming effect on children (Romeo et al., 2021). Moreover, individuals’ perceptions of mask wearers change over time. As previously mentioned, since the outbreak of the COVID-19 pandemic, individuals have tended to rate the health of mask wearers more positively (Miyazaki and Kawahara, 2016; Kamatani et al., 2021). However, there are limited studies in this field, and more research is needed in the future. Here are some of our proposed research ideas: (1) to examine the effect of face masks printed with different patterns on the attractiveness of faces; (2) to explore the judgment of different cultural groups on the trustworthiness of faces with different cultural masks; (3) to study the effect of facemasks of different colors on the degree of unhealthy initiation of people.

It is advised to investigate how face masks affect interpersonal perception in atypical groups (children/elders/patients). Most of the studies recruited young and healthy participants (Miyazaki and Kawahara, 2016; Freud et al., 2020; Olivera-La Rosa et al., 2020; Marini et al., 2021; Langbehn et al., 2022), but the effects of masks in atypical people, who are in the stages of growth or functional deterioration respectively, may be greater than those on young and healthy people. As mentioned earlier, face masks impede facial recognition in children (Roberson et al., 2012; Schneider et al., 2022). However, most of these studies were conducted at the beginning of an infectious disease outbreak (COVID-19), when child participants only lived in mask-wearing interactions environments for short periods. Children who are exposed to mask socialization for an extended period may have greater detrimental impacts. In contrast, in the elderly population, research has shown that face masks exacerbate the negative impact on interpersonal perception (Grundmann et al., 2021). Another study discovered that after one month of mandatory mask-wearing, elderly people became much more dependent on other visual cues (Chládková et al., 2021). Current research in the field has focused even less on unhealthy groups. However, the impact of face masks on the interpersonal perceptions of some patients may be even more severe, such as Epilepsy (Gomez-Ibañez et al., 2014), Depression (Douglas and Porter, 2010), Anxiety disorders (McClure et al., 2003), Alcohol dependence (Dethier et al., 2014), and Schizophrenia (Haut et al., 2010). Future research can improve the methods of previous studies, giving more theoretical direction and corresponding human care for enhancing social activities for atypical people when wearing masks.

## Author contributions

JC, XL, CH, and SW developed the study concept. SW and CH wrote the first version of the paper. ZS, XZ, SC, HW, GW, YX, XL, and JC provided critical revisions. All authors contributed to the article and approved the submitted version.

## Funding

This study was supported by the Natural Science Foundation of Zhejiang Province (LQ23C090004).

## Conflict of interest

The authors declare that the research was conducted in the absence of any commercial or financial relationships that could be construed as a potential conflict of interest.

## Publisher’s note

All claims expressed in this article are solely those of the authors and do not necessarily represent those of their affiliated organizations, or those of the publisher, the editors and the reviewers. Any product that may be evaluated in this article, or claim that may be made by its manufacturer, is not guaranteed or endorsed by the publisher.
